# ESKAPE Pathogens: Looking at Clp ATPases as Potential Drug Targets

**DOI:** 10.3390/antibiotics11091218

**Published:** 2022-09-08

**Authors:** Tehrim Motiwala, Qiniso Mthethwa, Ikechukwu Achilonu, Thandeka Khoza

**Affiliations:** 1 Discipline of Biochemistry, School of Life Sciences, University of Kwa-Zulu Natal-Pietermaritzburg Campus, Scottsville 3209, South Africa; 2 Protein Structure-Function Research Unit, School of Molecular and Cell Biology, University of the Witwatersrand, Johannesburg 2050, South Africa

**Keywords:** ESKAPE pathogens, antibiotic resistance, caseinolytic proteins, Clp ATPases

## Abstract

Bacterial antibiotic resistance is rapidly growing globally and poses a severe health threat as the number of multidrug resistant (MDR) and extensively drug-resistant (XDR) bacteria increases. The observed resistance is partially due to natural evolution and to a large extent is attributed to antibiotic misuse and overuse. As the rate of antibiotic resistance increases, it is crucial to develop new drugs to address the emergence of MDR and XDR pathogens. A variety of strategies are employed to address issues pertaining to bacterial antibiotic resistance and these strategies include: (1) the anti-virulence approach, which ultimately targets virulence factors instead of killing the bacterium, (2) employing antimicrobial peptides that target key proteins for bacterial survival and, (3) phage therapy, which uses bacteriophages to treat infectious diseases. In this review, we take a renewed look at a group of ESKAPE pathogens which are known to cause nosocomial infections and are able to escape the bactericidal actions of antibiotics by reducing the efficacy of several known antibiotics. We discuss previously observed escape mechanisms and new possible therapeutic measures to combat these pathogens and further suggest caseinolytic proteins (Clp) as possible therapeutic targets to combat ESKAPE pathogens. These proteins have displayed unmatched significance in bacterial growth, viability and virulence upon chronic infection and under stressful conditions. Furthermore, several studies have showed promising results with targeting Clp proteins in bacterial species, such as *Mycobacterium tuberculosis*, *Staphylococcus aureus* and *Bacillus subtilis*.

## 1. Introduction

**Figure 1:**
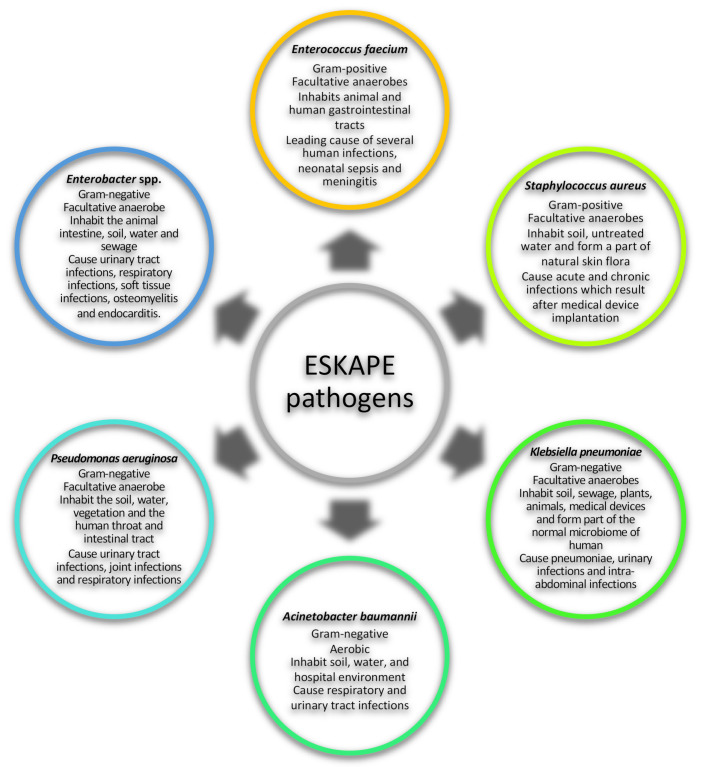
General characteristics ESKAPE pathogens. The ESKAPE group of pathogens consists of *Enterococcus faecium, Staphylococcus aureus, Klebsiella pneumoniae, Acinetobacter baumannii, Pseudomonas aeruginosa* and *Enterobacter* [1,2,3,10,11,12,13,14,15,16,17,18,19,20,21].

The ESKAPE pathogens are a group of pathogens that pose a global health threat due to their ability to evade antibiotic biocidal effects. This group of pathogens is composed of both Gram-positive and Gram-negative bacterial species, namely: *Enterococcus faecium, Staphylococcus aureus, Klebsiella pneumoniae, Acinetobacter baumannii, Pseudomonas aeruginosa* and *Enterobacter* (Figure 1) [1,2,3]. ESKAPE pathogens are mainly responsible for nosocomial infections and these infections are defined as hospital-acquired infections (HAIs) that affect patients within 48 h of admission, 3 days of discharge or 30 days of an operation [4]. HAIs present challenges in care delivery and no institution in any country seems to have solved this challenging situation. As a result, ESKAPE pathogens are responsible for more than 40% of infections in intensive care units (ICU) and pose an economic burden, especially in low- and middle-income countries [5,6]. Over the years, an increasing number of pathogens have been reported to be antibiotic resistant as a result of the misuse and overuse of antibiotics globally [1,7]. ESKAPE pathogens exhibit drug resistance via numerous mechanisms, such as using enzymes to irreversibly cleave and therefore inactivate the drug, modifying the drug-binding site, decreasing drug permeability or increasing drug efflux to decrease drug accumulation, or by the production of biofilms (Table 1) [3,8]. The emergence of antibiotic-resistant pathogens renders drugs that were initially used to combat bacteria to be redundant and ineffective, therefore allowing bacteria to grow in the presence of high antibiotic concentrations [1,7]. Subsequently, it is important to find alternative targets to inhibit the growth and spread of pathogens [2,3,9]. One such target is a group of proteins referred to as caseinolytic proteins, which are found in a number of organisms and play an important role in maintaining protein homeostasis in the cell [9].

Antivirulence drugs that do not necessarily kill the bacterial cells, but prevent bacterial pathogenesis by targeting virulence traits in bacteria, can be used to combat the emergence of antibiotic-resistant pathogens [27,28]. The use of antivirulence strategies to kill pathogens is advantageous as it results in less evolutionary pressure, thus reducing the development of resistant strains [27]. In this approach, the anti-ESKAPE drug administered would interfere with bacterial virulence factors instead of growth pathways to cure disease, thus leading to the development of new strategies for the prevention and control of infections [7,29].

## 2. Caseinolytic Proteins: Classification, Function and Structure

Caseinolytic (Clp) proteins are found in bacteria, fungi, in the mitochondria of eukaryotes and in the chloroplast of plants [9]. Microorganisms use Clp proteins, which function as a complex of catalytic (ClpP) and regulatory subunits (further referred to as Clp ATPases), to perform a variety of vital roles in the cell, such as protein homeostasis and cellular stress response (Table 2). An imbalance in protein quality and quantity control due to cellular stress, such as heat shock, the presence of antibiotics or a change in pH leads to a build-up of proteins in the cell, which results in cell death [9]. In such stressful conditions, it is important for the cell to remove these unfolded proteins for cellular function and growth to continue [30].

Additionally, it has been found that Clp proteins play an important role in the pathogenecity and virulence of several pathogens [42,43,44]. Several studies have linked ClpP to one of the mechanisms *S. aureus* uses to evade phagocytosis, which is one of the defence mechanisms that the host uses to fight *S. aureus* infection. For example, Frees et al. [42] established that *S. aureus* mutants lacking ClpX (Clp ATPase) or ClpP exhibited decreased virulence in a murine skin abscess model [42,43]. ClpP regulates the *agr* locus, which is responsible for the production of effectors, such as the haemolytic factor α-hemolysin. This effector generates small pores in the phagocytotic cells, enabling the phagocytosed bacterium to escape from the immune cells into the host system, thus enhancing the bacterium’s virulence [42,43,44]. Furthermore, this model also showed that the activity of ClpX and ClpP was crucial for the iron-regulated surface determinant system. This system is important for *S. aureus* iron uptake, which is essential for the pathogen to survive in the host [45]. This phenomena of ClpP regulating proteins to escape phagocytosis during an immune response is also observed in other bacteria, such as *Listeria monocytogenes.* This pathogenic bacteria causes listeriosis and it contains both ClpP1 and ClpP2 [46]. Although the functional significance of ClpP1 is unknown, *L. monocytogenes* mutants lacking ClpP2 were found to be susceptible to the activity of host macrophages, as these bacteria lacked their usual haemolytic abilities [47]. Finally, ClpP in *Pseudomonas aeruginosa* regulates the expression of alginate, an exopolysaccharide that protects the pathogen [48]. Furthermore, the importance of Clp proteins in bacterial virulence has also been demostrated in *Streptococcus pneumonia*, where strains lacking the *clpP* gene were found to lose their ability to invade lung tissues and colonise the nasopharynx [49]. Therefore, considering the importance of Clp proteins in the survival of pathogens in various environments, these proteins could be targeted for the development of potential drugs to inhibit their growth.

### 2.1. Catalytic Subunit—ClpP

**Figure 2:**
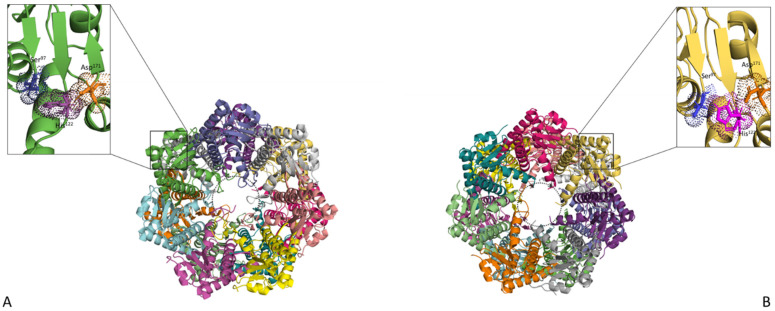
ClpP in its unbound and bound state. (**A**) Top view of the unbound ClpP tetradecamer (PDB—1YG6). (**B**) Top view of the active form of ClpP (PDB—5E0S). The Ser^97^, His^122^ and Asp^171^ catalytic residues are shown up-close and coloured orange, pink and blue, respectively. Upon Clp ATPase binding, ClpP assumes an opened/active conformation resulting in the ordering of the axial pore (represented by grey dotted lines) and the alignment of the catalytic residues. The structures were visualised using PyMol [58].

Most bacteria contain a single ClpP catalytic subunit with an exception of a few, such as *Mycobacterium tuberculosis*, *Mycobacterium smegmatis*, *Listeria monocytogenes*, *Chlamydia trachomatis* and *Pseudomonas aeruginosa*, which have been found to contain two ClpP homologs that are referred to as ClpP1 and ClpP2 [50,51,52,53,54,55]. ClpP is a serine protease composed of two heptameric rings, which forms a barrel-shaped structure referred to as a tetradecamer, and this tetradecamer may be formed by just one ClpP or a mixture of ClpP1 and ClpP2 homo- or hetero-tetradecamers. The ClpP tetradecamer encloses a protease active site with a catalytic triad consisting of three amino acids, namely serine, histidine and aspartic acid [56,57]. ClpP can adopt two conformations, that is: the closed/inactive and open/active conformation (Figure 2). In the closed conformation, the cavity for protein substrates to enter the proteolysis chamber is closed and the catalytic triad is misaligned. Therefore, protein hydrolysis is blocked. In this closed conformation, ClpP only functions as a peptidase, degrading only short peptides [36]. In order for ClpP to adopt an active conformation, it needs to be bound to a Clp ATPase (the regulatory subunit of a Clp protein) [36]. Clp ATPases bind to either one or both ends of ClpP; this binding results in conformational changes, which leads to the opening of the cavity for substrates to access the active site and in the alignment of the catalytic triad residues [43]. Subsequently, ClpP degrades damaged proteins, which are translocated into its chamber via Clp ATPases [36,56].

### 2.2. Regulatory Subunits—Clp ATPases

**Figure 3:**
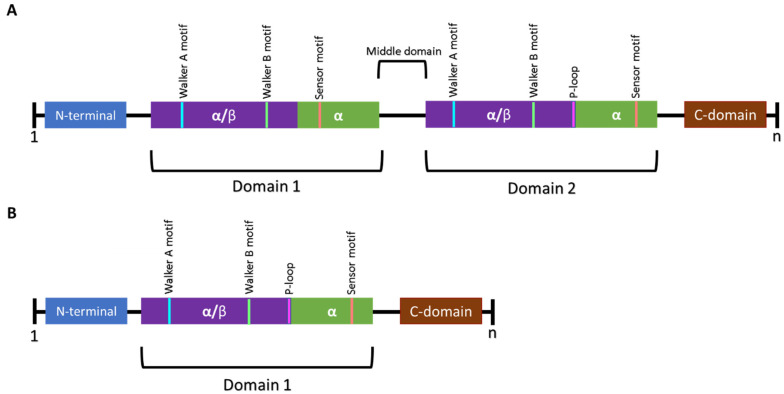
Schematic representation of the general structural features of Class I and Class II Clp ATPases of prokaryotes. (**A**) Class I Clp ATPases contain two nucleotide-binding domains (NBD) referred to as domain 1 and domain 2. A middle domain has been identified to be present in certain Clp ATPases and plays a role in separating the two NBDs. (**B**) Class II Clp ATPases contain one NBD. Each domain contains Walker A and Walker B motifs. Certain Clp ATPases contain a P-loop, a tripeptide (represented in pink) required for interaction with ClpP. n represents the number of amino acids in the sequence [60,61].

Clp ATPase proteins belong to a protein superfamily referred to as AAA+ proteins (ATPases associated with diverse cellular activities) [9]. The AAA+ ATPases have an AAA+ unfoldase/disaggregase, which recognises specific substrates and uses energy generated from ATP hydrolysis to contribute to functions, such as protein quality control, the degradation of transcriptional regulators, DNA replication and repair and cytoskeleton regulation, among other things [36]. The hallmark of the AAA family is a 200–250 amino acid ATP-binding domain that contains Walker A and Walker B motifs (Figure 3) [59]. The canonical Walker A forms the floor of the nucleotide-binding pocket (binds the ATP phosphate) and Walker B forms a loop that overlays the pockets and positions cations (binds metals and plays a role in ATP catalysis) [30,56]. Additionally, Clp ATPases have the unique ability to promote the resolubilisation of protein aggregates and are therefore grouped into the heat shock protein (Hsp) 100 family [9,30].

**Figure 4:**
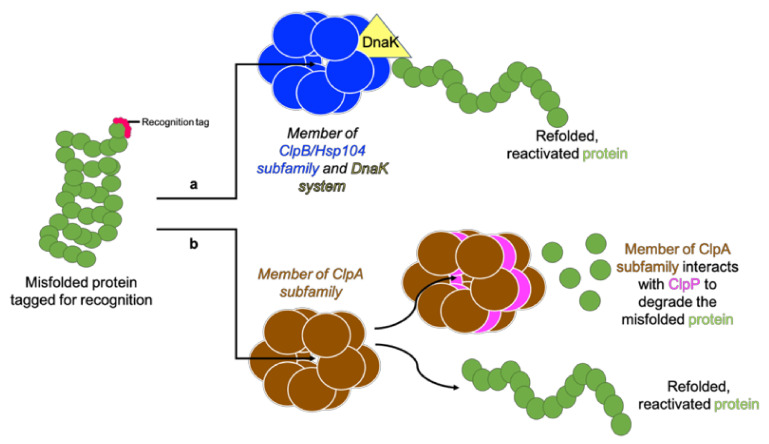
Two Clp ATPase subfamilies. A tagged protein is recognised by a Clp ATPase. (**a**) Members of the ClpB/Hsp104 subfamily lack the tripeptide for ClpP interaction and function along with the DnaK system to unfold and refold the protein into its functional conformation. (**b**) Members of the ClpA subfamily contain the tripeptide for ClpP interaction and therefore redirect proteins which cannot be unfolded and reactivated to ClpP for degradation. Adapted from Maurizi and Xia [61].

Clp ATPases are grouped into two classes (class I and II) based on the number of NBDs they contain. ClpA, ClpB, ClpC, ClpD, ClpE, ClpK and ClpL are class I members and contain two NBDs, whereas ClpM, ClpN, ClpX and ClpY have been identified as being class II members and contain one NBD (Figure 3) [39,56,62]. The Clp ATPases are further divided into two subfamilies, namely the ClpA and ClpB/Hsp104 subfamily based on the presence or absence of the tripeptide sequence [I-G-F/L] required for ClpP interaction, respectively (Figure 4) [36]. The ClpA subfamily forms hexameric complexes which bind and unfold proteins before translocating unfolded proteins to the ClpP proteolytic chamber for final degradation [9,36]. It is believed that the ClpB/Hsp104 subfamily functions as chaperones under stressful conditions to prevent protein unfolding or to assist in protein disaggregation as they lack the ClpP-binding motif [62,63]. Aligned with this chaperone activity, ClpB/Hsp104 have been reported to interact with the DnaK system to mediate protein unfolding and reactivation [39,62]. Members of both the ClpA and ClpB/Hsp104 subfamily have been identified in ESKAPE pathogens (Table 3).

## 3. Caseinolytic Proteins Targeted in ESKAPE Pathogens

To date, very few Clp proteins from ESKAPE pathogens have been identified (Table 3) and there are a limited number of drugs which are currently under investigation (Table 4). The current drugs being studied inhibit Clp proteins in one of three ways: firstly, they interfere with proteolytic activity by inhibiting ClpP; secondly, they interfere with ATPase activity by inhibiting or enhancing the activity of Clp ATPases; and lastly, they disrupt the ClpP and Clp ATPase complex [43,72,73]. Interestingly, of the drugs being studied, only five target *S. aureus*, which is a member of the ESKAPE pathogens and most of the other drugs target mainly *M. tuberculosis* (Table 4).

Drugs that have been developed to target the proteolytic subunit (ClpP) of Clp proteins in *S. aureus* include D2, E3 and G2 compounds (Table 4) which irreversibly inhibit the proteolytic activity of ClpP, therefore resulting in the build-up of damaged proteins in the cell and eventually leading to cell death [43]. Based on the current literature, ClpP associates with the ClpA subfamily to ultimately degrade proteins that cannot be unfolded and/or reactivated. Subsequently, targeting ClpP would prevent the formation of the ClpP-ClpA subfamily complex, thus impairing cell protein homeostasis [9].

**Figure 5:**
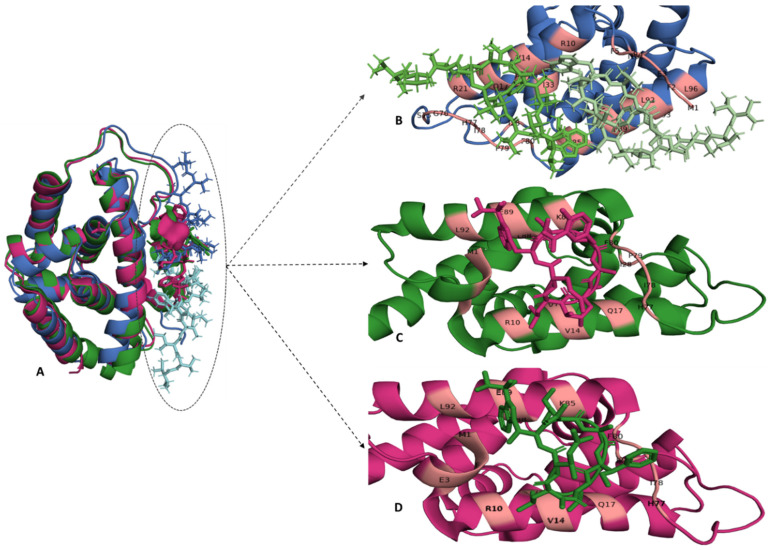
The binding site of Ecumicin, Cyclomarin-A and Rufomycin in the ClpC1 N-terminal domain. (**A**) The crystal structure of ClpC1-NTD-Ecumicin (PDB-6pbs), ClpC1-NTD-Rufomycin (PDB-6cn8) and ClpC1-NTD-Cyclomarin A (PDB-3wdc) are shown in blue, green and pink, respectively, and were superimposed to compare the local environment of the three ligands. (**B**) The binding of two ecumicin molecules (ecumicin 1-light green, ecumicin 2-dark green) per ClpC1 N-terminal domain shows that these molecules bind adjacent to each other. The binding stochiometric ratio of rufomycin (pink) or cyclomarin A (green) to ClpC1 N-terminal domain is 1:1, represented in (**C**) and (**D**), respectively. The binding sites for these anti-TB peptides are predominantly located on residues in α-helices 1 and 5 and the loop region connecting α-helices 4 and 5. The interacting residues are coloured light pink on their respective secondary structural elements. The structures and interactions were visualised using PyMol [58].

According to our knowledge, only one drug candidate, Armeniaspirols, targeting Clp ATPases belonging to the ClpB subfamily from *B. subtilis* has been reported. This drug candidate targets Clp ATPase (ClpY) when complexed to the proteolytic subunit (ClpQ) and is reported to disrupt the regulation of proteins essential for cell division, such as MreB and FtsZ [78]. On the other hand, the ClpA subfamily has a number of drug candidates being investigated, however most of these drug candidates target Mycobacterium tuberculosis, ClpC1 [Table 4]. The structures of lassomycin, cyclomarin A, rufomycin and ecumicin are show in Table 4 and these anti-TB peptides target the Clp ATPase subunit of Clp proteins, specifically the ClpC1 N-terminal domain (ClpC1-NTD) [16,43,74,76]. The three-dimensional structure of ClpC1-NTD complexed with some of these anti-TB peptides show that (a) their mode of action varies, (b) some may share a binding pocket (e.g., rufomycin cannot bind in the presence of ecumicin) and (c) the number of binding site/s on ClpC1-NTD varies per anti-TB peptide (Figure 5) [77,81,82]. For example, the crystal structure of ClpC1-NTD complexed with ecumicin or rufomycin showed that one rufomycin molecule binds to ClpC1-NTD, whereas two ecumicin molecules bind to ClpC1-NTD (Figure 5). The extent to which these anti-TB peptides impact the Clp ATPase is not similar across all of them, suggesting a difference in their mode of action. Biochemical studies have shown that ecumicin and lassomycin increase the ATPase activity of ClpC1, whereas rufomycin has no significant effect on ClpC1 ATPase activity [76,82,83,84]. Structural studies suggest that enhanced ClpC1 ATPase activity stimulated by ecumicin may be due to the conformational changes on the N-terminal domain and D1 interface, thereby improving ATP access for ATP hydrolysis in the nucleotide-binding domain [82]. As much as lassomycin binding to ClpC1 results in the overactivation of ClpC1 ATPase activity and therefore uncontrolled unfoldase activity [76], in the absence of ClpC1-NTD-lassomycin complex, it is unclear whether the binding of lassomycin to ClpC1 induces structural changes which are similar to those seen for ecumicin binding.

Given that lassomycin binds to an essential domain in Clp ATPases, it was interesting to observe that this compound is species specific, and is inactive against *S. aureus, Bacillus anthracis* and *K. pneumoniae.* One would expect functioning of Clp ATPases across different species to be impaired given that it targets a conserved region [30,76]. Keeping in mind the inability of designed compounds, such as lassomycin, to inhibit Clp ATPases across species, it may be worthwhile to design compounds to target specific motifs on Clp ATPases, ClpP and the ClpP/Clp–ATPase complex interface. It is unlikely that these compounds will be species- or class-specific but rather target Clp ATPases across species. This strategy will be advantageous since pathogens usually contain more than one Clp isoform, thus ensuring the complete inhibition of Clp activity in the pathogens [85]. There are a number of conserved motifs that could be targeted in Clp ATPases, these include the N-terminal domain, the extended N-terminal domain, the middle domain, the nucleotide-binding domain(s), the zinc-binding motif and the C-terminal domain [16,57]. These domains are responsible for a variety of functions. For example, the extended N-terminal domain of ClpB is important for the growth of bacteria at high temperatures in the absence of DnaK, whereas the extended N- terminal has been found to be important for homodimer formation in *Caenorhabditis elegans* ABCB6/HMT-1, suggesting that the N-terminal extension may be essential for Clp ATPase assembly and stability [86,87]. The primary function of the N-terminal domain is to stabilise Clp ATPases, therefore targeting the N-terminal domain would destabilise the Clp ATPases and lead to the loss of protein function [88].

The nucleotide-binding domain(s) and subsequently the Walker A and Walker B motifs are important drug targets, since drug candidates targeting this site would inhibit the ability of the Clp ATPase to hydrolyse ATP and consequently, the unfoldase activity of Clp ATPases will be abolished. We have noted a slight difference in the amino acid sequences of the NBDs between class I and class II, therefore it is important that drug-design studies are cognisant of this observation to ensure that the designed drugs are not class-specific. Another potential drug target is the middle domain and zinc-binding motif. The middle domain is implicated in protein stability and interdomain communication between NBD1 and NBD2. Taking into account, the importance of this domain in terms of protein stability, it is anticipated that targeting the middle domain would potentially destabilise the protein [89,90]. However, compounds developed against this region would need optimisation, given that the length of the middle domain differs across species and this domain is not observed to be in present in all Clp ATPases. The zinc-binding domain is present in ClpX and ClpK. The role of this domain in unknown in ClpK, however it is positioned in the N-terminal domain of ClpX and plays a role in substrate recognition [16,57,61]. Therefore, essentially compounds targeting the zinc-binding domain could inhibit substrate recognition and would theoretically lead to the build-up of damaged proteins in the cell.

## 4. Conclusions

ESKAPE pathogens are resistant to a number of antibiotics and pose a major threat to patients in the nosocomial environment, therefore it is important to develop alternative methods to target and control their spread. Clp proteins, which are essential for both virulence and survival of the pathogen, are emerging as potential drug targets. Therefore, these proteins have been studied in various organisms and a number of drug candidates are being investigated to target these proteins. As much as bioinformatic studies have shown that Clp proteins evolve to diversify their response to stressful environmental factors, certain key motifs and domains remain conserved. These conserved domains and motifs provide a potential site for the development of drug candidates to target Clp ATPases across species. Further studies could investigate the distribution of Clp proteins in ESKAPE pathogens and develop drug candidates that could reduce the impact of ESKAPE pathogens.

## Figures and Tables

**Table 1:** Resistance strategies used by ESKAPE pathogens to escape antibiotics.

Resistance Strategy	Resistance Mechanism	Antibiotics	ESKAPE Pathogens	References
**Drug** **inactivation**	Production of β-lactamase enzyme, which hydrolyses β-lactam rings	β-lactam (penicillin, carbapenems and	*K. pneumoniae*	[1,3,22]
		cephalosporins)	*P. aeruginosa, Enterobacter*	
	Contains chromosomally encoded AAC(6′)-Ii, which is responsible for enzymatic inactivation and EfmM ribosomal methylation	Tobramycin	*E. faecium*	[23]
	Carbapenemases, metallo-β-lactamases ^2^ and oxacillinase serine β-lactamases produced to catalyse antibiotic hydrolysis	Colistin, imipenem	*A. baumannii*	[3]
**Decreased drug influx**	Reduce the amount of porin protein OprD, or via loss of an outer membrane protein (Omp)	β-lactam (Imipenem and meropenem)	*P. aeruginosa, A. baumannii*	[3,23]
	Expresses enterococcal surface protein (ESP), which results in the formation of thicker biofilms	Vancomycin	*E. faecium*	[1]
	Thick cell wall traps and reduces antibiotic permeation	Vancomycin	*S. aureus*	[1]
	Mutation of *mbrB* gene	Colistin	*K. pneumoniae*	[24]
	Outer membrane (OmpF) protein with exclusion limit	MDR ^1^	*P. aeruginosa*	[1]
	Developing 4 resistant nodulation division (RND) type MDR efflux pump to remove toxic compounds from the periplasm and cytoplasm	MDR ^1^	*P. aeruginosa*	[3]
**Efflux pump system**	Nor-like efflux pump	Hydrophilic fluoroquinolones	*E. faecium*	[23]
	Expression of Penicillin-binding proteins (PBPs)	β-lactam (Penicillin, Cephalosporins, Carbapenems)	*S. aureus*	[1,3]
		Cephalosporins and aminoglycosides	*E. faecium*	[23]
	Upregulation of MexAB-OprM	Sulfonamides, cephalosporins, β-lactams, fluoroquinolones	*P. aeruginosa*	[1]
	AcrAB-TolC	Tetracyclines (including tigecycline)	*K. pneumoniae*	[25]
	Alteration of terminal sequence of cell wall precursors	VanA	*E. faecium*	[1]
**Drug site** **modification**	Expresses *mecA*, which encodes a low-affinity penicillin-binding protein	β-lactam (Penicillin, Methicillin)	S. aureus	[1]
	Expression of Aminoglycoside-modifying enzymes	Aminoglycosides	*P. aeruginosa*	[26]
	Qnr acts as a DNA homologue to compete for the DNA-binding site of DNA gyrase and topoisomerase IV	Quinolone	*K. pneumoniae*	[25]

^1^ Multidrug resistant: display non-susceptibility to at least one agent in three or more antimicrobial categories [2]. ^2^ Also known as imipenem metallo-β-lactamases (pathogens expressing these lactamases are able to escape the mechanism of action of imipenems) [3].

**Table 2:** Caseinolytic proteins identified across various species, exhibiting diverse functions.

Clp Catalytic Subunit			
	Species	Functions	References
ClpP	A number of bacteria including *Escherichia coli, Bacillus subtilis, S. aureus*	Proteolysis of damaged or misfolded proteins	[31]
**Clp regulatory subunit ^1^**			
ClpA ^2^	Gram-positive Proteobacteria	Protein quality control	[32]
ClpB	Prokaryotes, yeast, and plants	Disaggregation of stress-damaged proteins	[33,34,35]
	*Porphyromonas gingivalis*	Intracellular replication and survival	[36]
ClpC	Gram-positive bacteria (Firmicutes and Actinobacteria) and Cyanobacteria	Protein quality control, red blood cell lysis, regulate expression of virulence factors	[32,37]
ClpD	Chloroplasts of higher plants	Molecular chaperone	[35]
ClpE	Firmicutes	Thermotolerance, cell division and virulence	[32]
ClpK	*K. pneumonia*	Thermotolerance	[38]
ClpL	*Streptococcus pneumoniae*	Nucleotide phosphohydrolase activity, stabilises unfolded proteins, prevents protein aggregation	[39]
ClpV	Gram-negative bacteria	Component of the type V1 secrection system	[40]
ClpM	*Mus musculus*	Protein quality control	[35,41]
ClpN	*Pseudomonas aeruginosa*	Cell division	[35,41]
ClpX	Proteobacteria, Firmicutes and Thermatogae	Protein quality control, cell division, heat tolerance and virulence	[32,36]
ClpY	Gram-positive Proteobacteria	Cell division, heat shock response and capsule transcription	[32]

^1^ A number of Clp ATPases have been identified across various species and are named according to the species in which they have been identified, for example ClpK is from the *Klebsiella* species and ClpC1 is from *Mycobacterium tuberculosis*. ^2^ ClpA and ClpC are orthologs; bacteria usually contain either one of these [32].

**Table 3:** Caseinolytic proteins identified in ESKAPE pathogens.

ESKAPE Pathogens	Caseinolytic Proteins	References
*E. faecium*	ClpP	[64,65]
	ClpC	[64,66]
*S. aureus*	ClpP, ClpB, ClpC	[36,63]
	ClpX	[36,63]
*K. pneumoniae*	ClpK	[16,38,67]
*A. baumannii*	ClpP	[68,69]
*P. aeruginosa*	ClpXP and ClpP2	[48,70]
	ClpP	[38]
	ClpG	[71]
*Enterobacter*	None reported	

**Table 4:** Compounds developed as potential targets for Clp ATPases.

Compound	Structure ^1^	Mechanism of Action	References
**334**	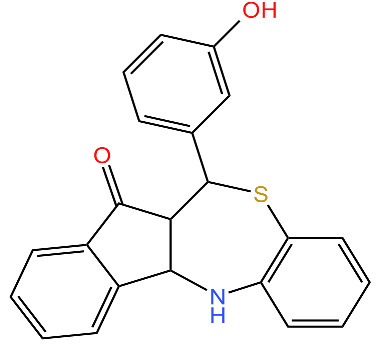	Deoligomerization of *S. aureus* ClpX, disrupts the ClpXP complex and blocks ClpX ATPase activity. *S. aureus* produces lower levels of toxins, such as hemolysins in the presence of the compound.	[43,74]
**D3**	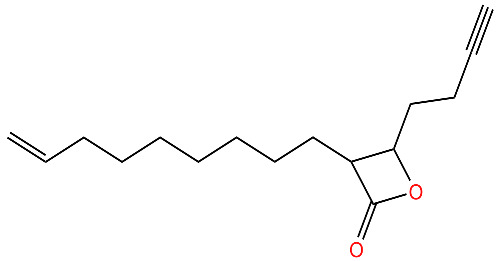	Irreversibly inhibits ClpP in methicillin-resistant *S. aureus*. Most potent inhibitor.	[43]
**E2**	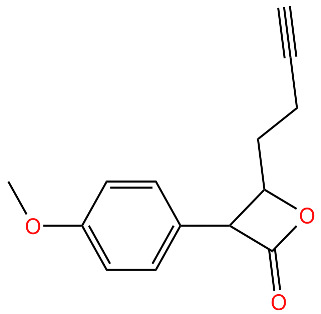	Irreversibly inhibits ClpP in methicillin-resistant *S. aureus*	[43]
**G2**	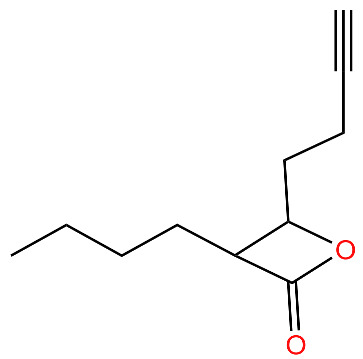	Irreversibly inhibits ClpP in methicillin-resistant *S. aureus*	[43]
**Acyldepsipeptides (ADEPs)**	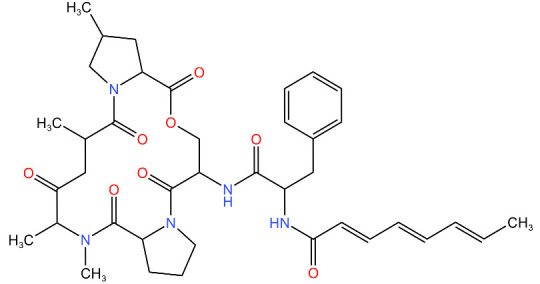	Prevents complex formation between ClpP and Clp ATPases in Gram-positive bacteria, such as *Enterococci* and *S. aureus*	[43,72,73]
**Ecumicin**	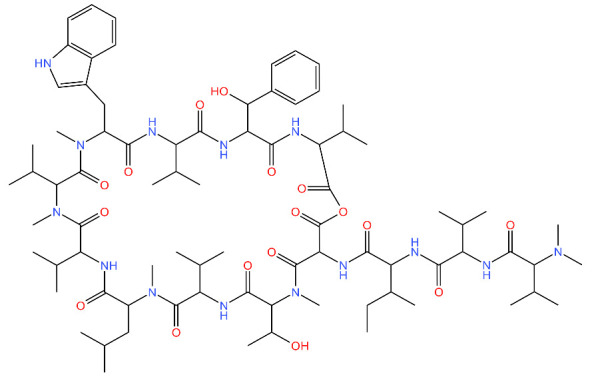	Binds to the N-terminal domain of ClpC1 of *M. tuberculosis.* Stimulates the ATPase hydrolysis activity of *M. tuberculosis* ClpC1 and at the same time decouples ClpC1 and ClpP, therefore inhibiting proteolytic activity and resulting in cell death.	[43,75]
**Cyclomarin A**	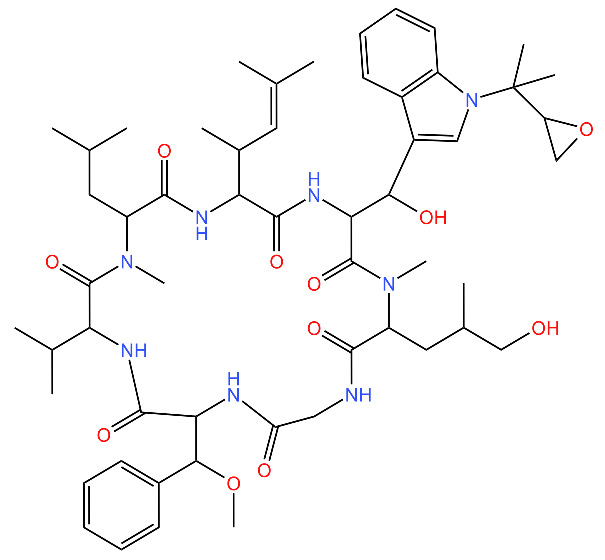	Binds to the N-terminal domain of ClpC1 of *M. tuberculosis* and prevents the movement of the N-terminal domain. Causes excessive proteolysis.	[43,76,77]
**Lassomycin**	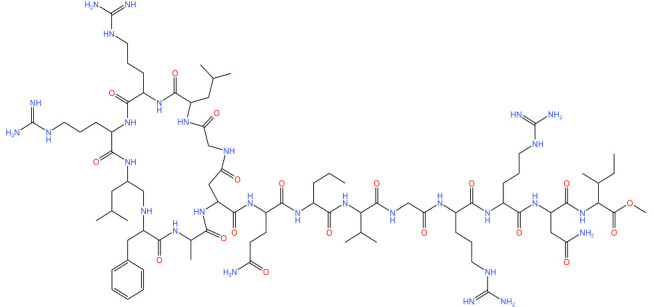	Binds to an acidic N-terminal pocket on ClpC1. Stimulates ATPase activity of ClpC1 from *M. tuberculosis,* however it also inhibits ATP-dependent degradation of proteins. Uncouples ClpC1 from ClpP1 and ClpP2, resulting in the death of the cell as unnecessary proteins build up.	[16,76]
**Rufomycin**	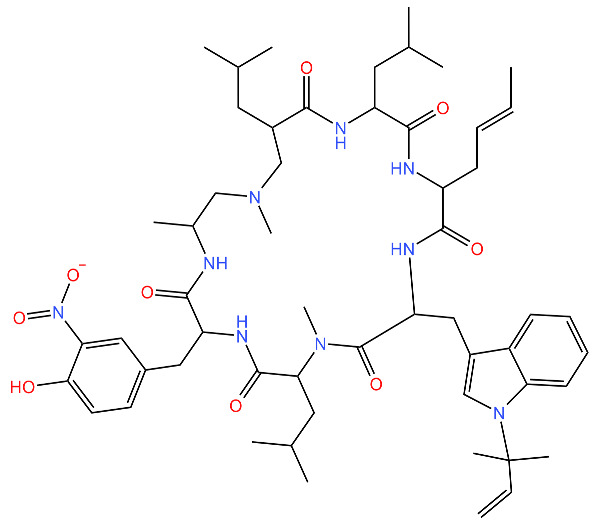	Interacts with the N-terminal domain of ClpC1 of *M. tuberculosis.* Decreases the proteolytic activity of the ClpC1 and ClpP complex, therefore resulting in the build-up of proteins in the cell.	[77]
**Armeiaspirols**	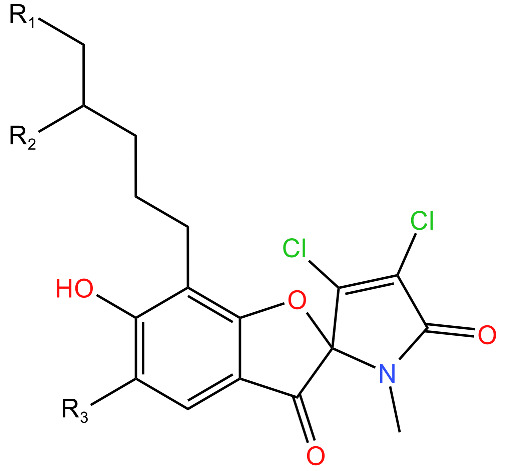	Inhibits ClpXP and ClpYQ in *Bacillus subtilis* by binding to the ATPase domains and therefore inhibits the function of the complexes. Inhibits ATP hydrolysis and proteolysis.	[78]
**Hydantoin analog**	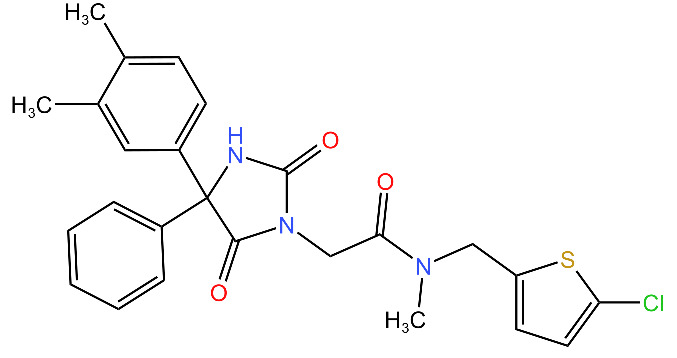	Inhibits the ClpXP complex. Binds to a binding pocket on ClpP and impairs complex substrate turnover.	[79]

^1^ Structures drawn using MarvinJS [80].
